# Effect of the Data Collection Method on Mobile Phone Survey Participation in Bangladesh and Tanzania: Secondary Analyses of a Randomized Crossover Trial

**DOI:** 10.2196/38774

**Published:** 2023-04-20

**Authors:** George Pariyo, Ankita Meghani, Dustin Gibson, Joseph Ali, Alain Labrique, Iqbal Ansary Khan, Gulam Muhammed Al Kibria, Honorati Masanja, Adnan Ali Hyder, Saifuddin Ahmed

**Affiliations:** 1 Makerere University School of Public Health, Makerere University College of Health Sciences Kampala Uganda; 2 Johns Hopkins Bloomberg School of Public Health Baltimore, MD United States; 3 Johns Hopkins Berman Institute of Bioethics Baltimore, MD United States; 4 Institute of Epidemiology, Disease Control and Research Dhaka Bangladesh; 5 Ifakara Health Institute Dar es Salam United Republic of Tanzania

**Keywords:** mobile phone survey, interactive voice response survey, non-communicable disease surveillance, response rate, cooperation rate, phone, risk, survey, public health, interview, voice, response, cooperation, female, women, rural, school, countries, non-communicable disease, surveillance, interactive survey

## Abstract

**Background:**

Mobile phone surveys provide a novel opportunity to collect population-based estimates of public health risk factors; however, nonresponse and low participation challenge the goal of collecting unbiased survey estimates.

**Objective:**

This study compares the performance of computer-assisted telephone interview (CATI) and interactive voice response (IVR) survey modalities for noncommunicable disease risk factors in Bangladesh and Tanzania.

**Methods:**

This study used secondary data from a randomized crossover trial. Between June 2017 and August 2017, study participants were identified using the random digit dialing method. Mobile phone numbers were randomly allocated to either a CATI or IVR survey. The analysis examined survey completion, contact, response, refusal, and cooperation rates of those who received the CATI and IVR surveys. Differences in survey outcomes between modes were assessed using multilevel, multivariable logistic regression models to adjust for confounding covariates. These analyses were adjusted for clustering effects by mobile network providers.

**Results:**

For the CATI surveys, 7044 and 4399 phone numbers were contacted in Bangladesh and Tanzania, respectively, and 60,863 and 51,685 phone numbers, respectively, were contacted for the IVR survey. The total numbers of completed interviews in Bangladesh were 949 for CATI and 1026 for IVR and in Tanzania were 447 for CATI and 801 for IVR. Response rates for CATI were 5.4% (377/7044) in Bangladesh and 8.6% (376/4391) in Tanzania; response rates for IVR were 0.8% (498/60,377) in Bangladesh and 1.1% (586/51,483) in Tanzania. The distribution of the survey population was significantly different from the census distribution. In both countries, IVR respondents were younger, were predominantly male, and had higher education levels than CATI respondents. IVR respondents had a lower response rate than CATI respondents in Bangladesh (adjusted odds ratio [AOR]=0.73, 95% CI 0.54-0.99) and Tanzania (AOR=0.32, 95% CI 0.16-0.60). The cooperation rate was also lower with IVR than with CATI in Bangladesh (AOR=0.12, 95% CI 0.07-0.20) and Tanzania (AOR=0.28, 95% CI 0.14-0.56). Both in Bangladesh (AOR=0.33, 95% CI 0.25-0.43) and Tanzania (AOR=0.09, 95% CI 0.06-0.14), there were fewer completed interviews with IVR than with CATI; however, there were more partial interviews with IVR than with CATI in both countries.

**Conclusions:**

There were lower completion, response, and cooperation rates with IVR than with CATI in both countries. This finding suggests that, to increase representativeness in certain settings, a selective approach may be needed to design and deploy mobile phone surveys to increase population representativeness. Overall, CATI surveys may offer a promising approach for surveying potentially under-represented groups like women, rural residents, and participants with lower levels of education in some countries.

## Introduction

Over the past couple of decades, the prevalence of noncommunicable diseases has increased, especially in low and middle-income countries [1,2]. These countries are also currently dealing with a double disease burden, with simultaneously higher prevalences of both communicable and noncommunicable diseases [3]. Regularly monitoring the prevalence and trends of these diseases is therefore crucial to direct prevention, treatment, and control efforts [2,4,5]. Household surveys conducted every 3 to 5 years, by the Demographic and Health Survey for example, are commonly used to collect representative population level health data in low and middle-income countries [6] and have been critical to tracking the progress of health interventions. To help monitor and address the rise of noncommunicable diseases, the World Health Organization launched the STEPwise approach to surveillance of noncommunicable disease surveys, which are currently the main source of nationally representative population estimates of noncommunicable disease prevalence and behavioral risk factors in low and middle-income countries [7]. However, conducting such surveys is expensive, time-consuming, and labor-intensive [8,9].

In high-income countries, population-level health and demographic data are routinely collected through landline telephone surveys, such as the Behavioral Risk Factor Surveillance System in the United States [10]. Participation in landline-based surveys has declined as households become increasingly reliant on mobile phones as their primary mode of connection [11,12]. Although the differences in ownership and subscription of mobile phones between high-income countries and low or middle-income countries have been large, the rates have become similar in recent years. More than two-thirds of the world’s mobile phone subscribers reside in low and middle-income countries [13]. With the promise of gathering real-time or more frequent survey data on population health, as well as informing decisions on programs and priorities at potentially lower cost and time, mobile phone surveys have the potential to provide complementary population-level data on noncommunicable disease risk factors [14-18]. Moreover, populations living in remote and hard-to-reach areas could become more accessible, potentially enabling faster and more timely collection of public health surveillance data from them [19]. Several mobile phone data collection methods, such as computer-assisted telephone interviews (CATIs), interactive voice response (IVR), and SMS text messaging, are in use in many low and middle-income countries [20]. With CATI surveys, human interviewers at call centers administer questions following a standard script with the help of a computer and capture responses directly into electronic format on a computer, tablet, or other devices. With IVR surveys, respondents interact with a preprogrammed digital audio interview, which contains both questions and a series of predetermined answers to the questions that are linked to a numeric response on a touch-tone phone keypad (eg, “Press 1 for Yes”). SMS text messaging surveys operate in a similar manner but through the exchange of textual information [19,21,22]. Although these data collection methods are in use, the most effective sampling methodologies or mechanisms for deploying mobile phone surveys are not yet well understood.

Data collection using mobile phone surveys is still in its infancy, and before implementing it at a large scale or before thinking about replacing household surveys, more research is required to improve participation. For instance, previous mobile phone surveys obtained samples different from household surveys or nationally representative estimates [21,23]. Some studies have shown that using different consent approaches or incentives may improve survey participation [17,21,24]. Studies implemented in one country may differ from studies in other countries. Furthermore, there is a paucity of published literature comparing survey representativeness and other aspects of data quality across different mobile phone survey modalities in low and middle-income countries [19,25], and relatively little data exist on key parameters affecting mobile phone survey outcomes, including contact, response, completion, refusal, and cooperation rates in low and middle-income countries. This lack of knowledge limits our understanding of the factors to consider for the evidence-based design of mobile phone surveys.

Using data from 2 low and middle-income countries, Bangladesh and Tanzania, this study compared the performance of CATI and IVR modalities on selected key survey metrics: contact, response, cooperation, completion, and refusal rates. Investigating these factors is critical for designing and implementing mobile phone surveys that can achieve population representativeness and high survey quality during data collection. This will also help to understand the feasibility, cost, and time required for collecting data from a representative sample.

## Methods

### Survey Design

This secondary analysis was performed with the data from a randomized crossover trial. The trial was conducted to enable a test-retest assessment of response consistency and reliability, allowing for a 7-day gap between the CATI and IVR surveys [26]. Although the parent study used a test re-test design of collecting data from the *same individuals* using 2 modes, in this manuscript, we only included the initial independent samples of *different individuals*, each reached using only either of the 2 modes, CATI or IVR. Also, unlike the parent study, which focused on reliability, this paper specifically examined 4 indicators: survey contact, response, refusal, and cooperation rates between those who received the CATI surveys and those who received the IVR surveys at the first contact only, hereafter referred to as the CATI and IVR arms, respectively.

### Participants

Study participants in Bangladesh and Tanzania were identified using a random digit dialing approach. Phone number prefixes (2-4 digits) unique to each mobile network operator registered and active in Bangladesh and Tanzania were identified. Using these unique prefixes, the remaining 6 to 8 digits were randomly generated via a computer for each country to create large pools of unique mobile phone numbers to which the CATI and IVR surveys were to be delivered [15]. In both countries, we chose larger starting samples for IVR than for CATI because initial pilot tests using IVR surveys showed more calls were required to obtain a completed response rate than with CATI surveys, although the extent of this difference was unknown.

### Randomization

In each country, 2 study arms were created: CATI and IVR. Based on the modality assigned, a survey call was placed using either CATI (human caller) or IVR (automated). To recruit respondents, the individual who answered the phone became the study participant if they indicated that they were at least 18 years old and provided their consent to participate in the survey. For respondents younger than 18 years or who did not provide consent, the call was ended immediately but noted for subsequent analysis of case dispositions. Further details on the methods used in this study are contained in the published protocol [22].

### Procedure

The questions used for the CATI and IVR surveys were the same and included a demographic module and noncommunicable disease modules. Though previous studies showed differences in participation rates with these 2 survey modalities, we kept the same set of questions to compare the responses. However, as expected, there could be a difference in participation due to a real voice and recorded voice in the CATI and IVR surveys, respectively. We did not check whether the interviewer’s voice and survey mode had any interaction. We defined a noncommunicable disease module as a group of domain-specific, related questions pertaining to noncommunicable disease risk factors, including tobacco use, alcohol intake, consumption of fruits and vegetables, physical activity, and use of health care screening and treatment for selected noncommunicable diseases [22]. Questionnaires used in the study have previously been published [26]. The survey introduction and response options were adjusted for the survey modality. The same technology platform (ie, Viamo) was used to deliver IVR surveys in both countries. For the CATI surveys, enumerators in each country were drawn from those already trained and experienced in CATI methodology. Additionally, the research team trained enumerators on best practices for CATI (eg, survey data capture and documentation), the contents of the noncommunicable disease risk factor survey, and essential aspects of research ethics.

Respondents in all surveys did not incur any charges for receiving calls, and small airtime credits were delivered to their prepaid mobile phone number upon completion of the survey. The amount, timing, and structure of the incentive provided were based on information obtained from a related study [21]. To mitigate the possible effect of time of day on responses, calls for both CATI and IVR surveys were randomly distributed and placed between 8 AM and 8 PM local time in both countries. If the targeted respondent missed or disconnected the initial call, the CATI enumerator or IVR platform made 3 additional attempts to the same number [27]. Calls were made available in Bangla and English in Bangladesh and in Kiswahili and English in Tanzania. Respondents could select the preferred survey language by telling the enumerator at the start of CATI surveys or pressing a number on their telephone keypad for IVR. Data collection lasted from the end of June 2017 to mid-July 2017 in Tanzania and mid-August 2017 to the end of August 2017 in Bangladesh.

### Survey Outcomes

The definition of each outcome is presented in Table S1 in Multimedia Appendix 1. Response rates were calculated using standard definitions from the American Association for Public Opinion Research [28], with minor adaptations to accommodate the random digit dial selection of mobile phone numbers. We calculated primary survey response rates using a conservative scenario in which denominators for contact, response, and refusal rates included all the known (ie, calls connected), and unknown (ie, numbers that did not connect and remained unknown as to their assignment status or eligibility) phone numbers. In addition, we calculated survey response rates using a realistic scenario for the denominators for contact, response, and refusal rates by including only the known or confirmed mobile phone numbers (ie, those who picked up or calls that connected) plus only an estimated proportion of unknowns (*e*) who could be expected, on the basis of survey data, to be eligible. Denominators for cooperation rates in both primary and secondary analyses did not include any unknowns. We considered a complete interview to be a survey in which the respondent answered the demographic module and at least 4 of 5 noncommunicable disease modules. Partial interviews were those in which the respondent completed the demographic module and answered at least one question in any of the noncommunicable disease modules. Our operational definitions of the survey dispositions are included in Table S2 in Multimedia Appendix 1.

### Sample Size

Sample size calculations were performed based on a crossover design for the parent study in which the present study was nested [26]. The completion rate estimates were obtained during country adaptation and technology testing trials [22]. Assuming a baseline survey completion percentage of 30%, in order to detect an absolute 10% difference in survey completion between the 2 study arms at an alpha of .05 and power of 80%, we calculated that 376 *completed* mobile phone surveys were needed in each study arm. Adjusting for the expected completion percentage of 30%, we calculated that 1254 participants needed to *consent* to the survey per study arm.

### Data Analysis

We conducted the data analysis under the null hypothesis that respondents of CATI and IVR surveys are not significantly different in terms of sex, age, schooling, and rural or urban residence. In addition, under this assumption of no difference between respondents to the 2 modes, we would not expect to see differences in survey completion, contact, response, refusal, and cooperation rates between those who were contacted for the CATI survey and those who were contacted for the IVR survey.

First, we reported the sociodemographic characteristics (ie, age, sex, location, and education) of the survey participants by survey mode and country. We also conducted logistic regression analysis to report the unadjusted odds ratio (UOR), adjusted odds ratio (AOR), and 95% CI for participating in the IVR survey compared with participating in the CATI. Next, we reported the survey rates (ie, contact, response, refusal, and cooperation rates) by survey mode. The samples are presented alongside the latest available census report from both countries [29,30].

We fit multilevel, multivariable logistic regression models using the *gllamm* module [31] for each country to estimate the association of survey mode with main survey outcomes (ie, response rate, cooperation rate, complete interview, and partial interview). Models fitted using *gllamm* have the feature of combining fixed effects and potential or latent random effects due to clustering and other unknown factors specific to the mobile network platforms or plans through which calls were received. Level 1 units were the individual calls, and level 2 units were the mobile phone network plans.

For each outcome, in addition to survey mode, we included the following covariates in each model: age, sex, location of residence, and education level. Data analysis was performed with Stata 14.0 SE (StataCorp LLC, College Station, TX).

### Ethical Approval

Ethical approval was received from the institutional review boards (IRBs) at Johns Hopkins Bloomberg School of Public Health, Baltimore, Maryland (IRB protocol number 00007318); the Institute of Epidemiology, Disease Control and Research in Bangladesh (IRB protocol number IEDCR/IRB/2016/14); and the Ifakara Health Institute and National Institute of Medical Research in Tanzania (IRB protocol number IHI/IRB/No. 036-2016). Since all contact with participants was through their mobile phones, an adapted oral consent was used for CATI, and the same script was modified for IVR delivery [18,32].

## Results

### Sample Characteristics

A total of 67,907 random digit dialed calls were placed in Bangladesh for both modes, and a total of 56,084 random digit dialed calls were placed in Tanzania for both modes. The survey case dispositions by mode for each country are shown in Table 1.

Self-reported data on demographic characteristics are shown in Table 2. The respondents tended to be younger (ie, 18-29 years old), men, urban residents, and relatively higher educated. Those in the age category of 30-49 years appeared to be less likely to participate in IVR surveys compared with the age category of 18-29 years in both Bangladesh (UOR=0.52, 95% CI 0.40-0.69) and Tanzania (UOR=0.41, 95% CI 0.31-0.55). Respondents aged 50 years old were more likely to participate in IVR surveys in Bangladesh (UOR=2.17, 95% CI 1.38-3.51), while the result was opposite to that in Tanzania (UOR=0.31, 95% CI 0.20-0.49). Although the proportion of women was higher among CATI respondents than among IVR respondents in both countries, comparisons across the 2 survey modes showed that men were significantly more likely than women to respond to IVR surveys in both Bangladesh (UOR=3.87, 95% CI 2.84-5.25) and Tanzania (UOR=1.58, 95% CI 1.20-2.10). Urban residents had higher odds of responding to IVR surveys than to CATI surveys in Bangladesh (UOR=3.87, 95% CI 2.84-5.25), but the odds were lower in Tanzania (UOR=0.73, 95% CI 0.55-0.95). Last, people who had attempted at least secondary education had higher odds in Bangladesh only. IVR and CATI surveys had a higher proportion of younger people, men, urban residents, and educated people than census residents in both countries.

**Table 1:** Case dispositions, as defined by the American Association for Public Opinion Research (AAPOR) with some adaptation (unknown number) for mobile phone surveys, and survey completion using computer-assisted telephone interviews (CATIs) and interactive voice response (IVR) mobile phone surveys in Bangladesh and Tanzania.

Call outcome and survey characteristics			Bangladesh					Tanzania		
			CATI		IVR		CATI			IVR
Total unique calls attempted, n			7044		60,863		4399			51,685
Status identified, n			949		1026		447			801
Complete interview (I), n			359		371		375			448
Partial interview (P), n			18		127		1			138
Refusal/withdrawal, n			572		285		67			114
**Reason for refusal or withdrawal, n**										
	Refused/no consent	572		120		67			32	
	Disconnected call	0		165		0			82	
Unknown phone numbers (UH^a^+UO^b^), n			6095		59,594		3948			50,783
Estimated proportion of unknown phone numbers expected to be eligible *I*			0.0631		0.0135		0.0872			0.0138
*e*(UH+UO)			385		803		3344			702
Total ineligible, n			6095		59,837		3952			50,884
**Reason for ineligibility, n**										
	Age	0		243		4			101	
	Unknown or nonexistent phone number	6095		59,594		3948			50,783	

**^a^**UH: unknown household or phone number.

^b^UO: unknown other.

**Table 2:** Characteristics of eligible respondents in Bangladesh and Tanzania to mobile phone surveys using computer-assisted telephone interviews (CATIs) and interactive voice response (IVR) for the first contact.

Characteristics		Bangladesh					Tanzania			
		CATI, n (%)	IVR, n (%)	UOR^a^ (95% CI; reference: CATI)	Distribution from last census, %	CATI, n (%)		IVR, n (%)	UOR (95% CI; reference: CATI)	Distribution from last census, %
Age (years), median (IQR)		27 (21-35)	25 (20-32)	N/A^b^	N/A	32 (25-42)		26 (22-32)	NA	N/A
**Age group (years)**										
	18-29	254 (57.1)^c^	542 (64.1)^d^	Reference	33	158 (41.8)^e^		470 (64.9)^f^	Reference	41
	30-49	159 (36.1)^c^	178 (21.1)^d^	0.52 (0.40-0.69)	47	167 (44.2)^e^		205 (28.3)^f^	0.41 (0.31- 0.55)	39
	≥50	27 (6.1)^c^	125 (14.8)^d^	2.17 (1.38-3.51)	20	53 (14.0)^e^		49 (6.8)^f^	0.31 (0.20- 0.49)	20
**Sex**										
	Female	162 (36.9)^g^	85 (13.1)^h^	Reference	50	137 (36.4)^i^		177 (26.6)^j^	Reference	51
	Male	277 (63.1)^g^	562 (86.9)^h^	3.87 (2.84-5.25)	50	239 (63.6)^i^		489 (73.4)^j^	1.58 (1.20-2.10)	49
**Location**										
	Rural	264 (60.3)^k^	257 (40.3)^l^	Reference	77	129 (34.3)^i^		274 (41.8)^m^	Reference	70
	Urban	174 (39.7)^k^	380 (59.7)^l^	2.24 (1.74-2.90)	23	247 (65.7)^i^		381 (58.2)^m^	0.73 (0.55-0.95)	30
**Level of education attempted**										
	No school	63 (14.3)^c^	59 (7.0)^d^	Reference	47^n^	21 (5.6)^e^		49 (6.8)^f^	Reference	8
	Primary	168 (38.2)^c^	134 (15.9)^d^	0.85 (0.55-1.33)	26	212 (56.1)^e^		304 (42.0)^f^	0.61 (0.34-1.08)	75
	Secondary	76 (17.3)^c^	190 (22.5)^d^	2.67 (1.67-4.26)	17	113 (29.9)^e^		254 (35.1)^f^	0.96 (0.52-1.73)	14
	At least university or tertiary	128 (29.1)^c^	246 (29.1)^d^	2.05 (1.33-3.17)	10	28 (7.4)^e^		7 (1.0)^f^	0.11 (0.03-0.31)	2
	Missing	5 (1.1)^c^	216 (25.6)^d^	N/A	N/A	4 (1.1)^e^		110 (15.2)^f^	N/A	N/A

^a^UOR: unadjusted odds ratio.

^b^N/A: not applicable.

^c^n=440.

^d^n=845.

^e^n=378.

^f^n=724.

^g^n=439.

^h^n=647.

^i^n=376.

^j^n=666.

^k^n=438.

^l^n=637.

^m^n=655.

^n^These participants were illiterate.

### Survey Response Rates

CATI surveys had a higher response rate in Bangladesh, at 5.4% (377/7044), than IVR surveys, at 0.8% (498/60,377). Similarly, in Tanzania, the response rates were 8.6% (376/4391) for CATI surveys and 1.1% (586/51,483) for IVR surveys (Table 3).

**Table 3:** Primary survey rates by mobile phone survey delivery mode in Bangladesh and Tanzania.

AAPOR^a^ category	Bangladesh			Tanzania	
	CATI^b^ (n=7044), n (%)	IVR^c^ (n=60,377), n (%)	CATI (n=4391), n (%)		IVR (n=51,483), n (%)
Contact rate #1	949 (13.5)	783 (1.3)	443 (10.1)		700 (1.4)
Response rate #2	377 (5.4)	498 (0.8)	376 (8.6)		586 (1.1)
Refusal rate #1	572 (8.1)	285 (0.5)	67 (1.5)		114 (0.2)
Cooperation rate #2	377 (39.7)^d^	498 (63.6)^e^	376 (84.9)^f^		586 (83.7)^g^

^a^AAPOR: American Association for Public Opinion Research.

^b^CATI: computer-assisted telephone interview.

^c^IVR: interactive voice response.

^d^n=949.

^e^n=738.

^f^n=443.

^g^n=700.

### Survey Response and Cooperation

We reported the results of multilevel logistic regression analysis to examine the adjusted association of response and cooperation rates with survey modality (Table 4). The odds of a response to (AOR=0.73, 95% CI 0.54-0.99) and cooperation with (AOR=0.12, 95% CI 0.07-0.20) IVR were lower in Bangladesh. The odds ratios were similar in Tanzania; however, other demographic factors (ie, age, gender, location, and education) were not significant in the adjusted model.

**Table 4:** Odds ratios (ORs) from the multilevel logistic regression modeling (generalized linear latent and mixed models; gllamm) for mode effects on primary survey response rates for computer-assisted telephone interview (CATI) and interactive voice response (IVR) mobile phone surveys in Bangladesh and Tanzania.

Variables			Bangladesh					Tanzania		
			Adjusted OR (95% CI)		*P* value		Adjusted OR (95% CI)			*P* value
**AAPOR^a^ response #2^b,c^**										
	IVR (reference: CATI)^d^	0.73 (0.54-0.99)		.04		0.31 (0.16-0.60)			.001	
	Older age (50-69 and ≥70 years; reference:18-49 years)	1.01 (0.55-1.85)		.98		0.87 (0.26-2.93)			.82	
	Female (reference: male)	0.71 (0.51-0.99)		.045		0.82 (0.42-0.61)			.56	
	Rural (reference: urban)	1.52 (1.14-2.03)		.004		1.54 (0.78-3.05)			.22	
	Lower level of education (none or primary only; reference: at least secondary)	16.13 (8.93-29.13)		<.001		0.89 (0.47-1.70)			.74	
**AAPOR cooperation #2^c,e^**										
	IVR (reference: CATI)^d^	0.12 (0.07-0.20)		<.001		0.28 (0.14-0.56)			<.001	
	Older age (50-69 and ≥70 years; reference:18-49 years)	1.02 (0.48-2.13)		.95		0.85 (0.25-2.88)			.80	
	Female (reference: male)	0.52 (0.33-0.81)		.002		0.88 (0.44-1.77)			.72	
	Rural (reference: urban)	1.36 (0.94-1.98)		.11		1.49 (0.75-2.95)			.26	
	Lower level of education (none or primary only; reference: at least secondary)	9.37 (4.84-18.13)		<.001		0.85 (0.44-1.63)			.62	

^a^AAPOR: American Association for Public Opinion Research.

^b^Bangladesh: level 1 n=1570 and level 2 n=5; Tanzania: level 1 n=1494; level 2 n=8. Level 1 units were the individual calls, and level 2 units were the mobile phone network plans.

^c^Rates and numbers indicate key survey rates as defined by the AAPOR.

^d^In this analysis, IVR was used in the first contact and is the primary survey mode; CATI used in the first contact is the counterfactual.

^e^Bangladesh: level 1 n=1453 and level 2 n=5; Tanzania: level 1 n=1493; level 2 n=8.

Secondary survey response and cooperation rates were higher than those in the primary analysis (Table S3 in Multimedia Appendix 1). Secondary analysis results largely showed a similar picture in the multilevel, multivariable analysis for mode effects and effects of age, gender, and education level on survey response and cooperation in both countries, except in Bangladesh, rural residence did not show an additional significant effect (Table S4 in Multimedia Appendix 1).

### Survey Completion and Partial Completion

Table 5 reports the odds of complete and partial interviews in both countries. Those who were contacted for IVR surveys were significantly less likely than those contacted for CATI surveys to complete the survey in both Bangladesh (AOR=0.33, 95% CI 0.25-0.43) and Tanzania (AOR=0.09, 95% CI 0.06-0.14). However, the AORs for partial interviews were higher both in Bangladesh (AOR=7.94, 95% CI 4.80-13.14) and Tanzania (AOR=15.12, 95% CI 8.99-25.44). The adjusted models in Bangladesh showed positive associations of male gender, rural location, and higher education with complete interviews and positive associations of higher education with partial interviews; however, these factors were not associated with partial or complete interviews in Tanzania.

**Table 5:** Odds ratios (ORs) from the multilevel logistic regression modeling (generalized linear latent and mixed models; gllamm) for complete and partial computer-assisted telephone interviews (CATIs) and interactive voice response (IVR) mobile phone surveys in Bangladesh and Tanzania.

Variables		Bangladesh			Tanzania	
		Adjusted OR (95% CI)	*P* value	Adjusted OR (95% CI)		*P* value
**Complete interviews^a,b^**						
	IVR (reference: CATI)^c^	0.33 (0.25-0.43)	<.001	0.09 (0.06-0.14)		<.001
	Older age (50-69 and ≥70 years; reference:18-49 years)	0.73 (0.47-1.14)	.16	0.65 (0.35-1.20)		.17
	Female (reference: male)	0.71 (0.53-0.96)	.02	0.72 (0.51-1.03)		.07
	Rural (reference: urban)	1.58(1.24-2.01)	<.001	0.97 (0.70-1.36)		.88
	Lower level of education (none or primary only; reference: at least secondary)	2.45 (1.88-3.19)	<.001	0.76 (0.55-1.06)		.11
**Partial interviews^b,d^**						
	IVR (reference: CATI)^c^	7.94 (4.80-13.14)	<.001	15.12 (8.99-25.44)		<.001
	Older age (50-69 and ≥70 years; reference:18-49 years)	1.62 (0.92-2.83)	.09	1.60 (0.82-3.13)		.17
	Female (reference: male)	1.25 (0.79-1.98)	.34	1.39 (0.94-2.05)		.10
	Rural (reference: urban)	0.78 (0.54-1.12)	.17	1.18 (0.82-1.69)		.38
	Lower level of education (none or primary only; reference: at least secondary)	2.62 (1.84-3.74)	<.001	1.34 (0.93-1.94)		.12

^a^Bangladesh: level 1 n=1570 and level 2 n=5; Tanzania: level 1 n=1494; level 2 n=8. Level 1 units were the individual calls, and level 2 units were the mobile phone network plans.

^b^Rates were as defined by the American Association for Public Opinion Research (AAPOR).

^c^In this analysis, IVR was used in the first contact and is the primary survey mode; CATI used in the first contact is the counterfactual.

^d^Bangladesh: level 1 n=1453 and level 2 n=5; Tanzania: level 1 n=1493; level 2 n=8.

## Discussion

### Principal Findings

This study evaluated the effect of CATI and IVR survey modes on some key survey metrics—contact, response, cooperation, completion, and refusal rates—and showed how these metrics vary with these 2 survey modes and key sociodemographic factors. We observed a relatively higher response, cooperation, and completion rates with CATI than with IVR. We also found that gender, education level, and residence had additional independent effects on survey response, cooperation, and completion rates in Bangladesh but not in Tanzania.

We found the CATI survey mode to be more likely to include respondents traditionally considered to be harder to reach through mobile phones such as women, those in rural areas, and those with lower levels of education [19,20]. Surprisingly, age did not appear to have an additional independent effect on top of survey delivery mode in both countries. Given the automated nature of IVR surveys, we expected that respondents who (1) live in urban areas, (2) are younger, and (3) have higher education levels would be more likely to respond to IVR surveys as compared with their counterparts. Similarly, we expected that CATI mode would yield more participation than IVR among the groups reported to be traditionally less likely to respond to automated surveys such as those in rural areas, older participants, and those with lower education levels. Previous studies suggest that greater ability to comprehend and process questions independently are required of IVR survey respondents, and therefore, the resultant effect of nonresponse tends to be more pronounced in respondents who are older or have lower levels of education [23,33]. In comparison, due to the “human” element of CATI surveys, in which respondents can interact with a live interviewer, we expected that respondents who were older, less educated, and rural residents would be more likely to respond to CATI compared with IVR surveys. CATI surveys have been known to increase response rates because of the reciprocity that exists between the interviewer and respondent, allowing respondents to clarify questions being asked, but also because of social norms, where respondents may find it difficult to hang up on a live interviewer once the interview commences [34].

Despite the reported lower percentages of urban than rural residents in both countries [35], we observed that, with the exception of CATI respondents in Bangladesh, most respondents reported being located in urban areas and were also primarily younger men, which is consistent with findings from other studies [36,37]. This may also be attributed to greater penetration of phones in urban areas [38,39], which increases access to these groups. In addition, possible inadequate access to electricity and cost of airtime of mobile phones might mean that mobile phones are frequently turned off or unreachable among rural participants [23].

The low participation may occur as some respondents may view surveys as an interruption of their time, intrusion on their privacy, or, more generally, automated “robocall” or spam calls [12,40]. In the United States, there are nearly 3.4 billion robocalls per month, and worldwide, the number of robocalls grew by 325% in 2018 [41]. Patterns of decreased participation in telephone and mobile phone surveys have already been documented in high-income countries and may similarly be observed in low and middle-income countries but require further exploration. Moreover, some apps and available services may also prevent them from receiving calls when these apps or services consider a number as spam [42,43]. Using a designated mobile phone number and establishing a hotline where participants can verify information about the phone calls may be helpful. In high-income countries with an established tradition of telephone surveys, there is mixed data on whether demographic and health-related results observed among participants of a landline telephone survey are comparable to those with mobile phones [36,44]. Most low and middle-income countries have “leap-frogged” the phase of landline telephone surveys, and it is difficult to find comparable data. With the expanding mobile phone service, it is important to increase the number of participants considering the opportunities provided by these surveys. More research is required to better understand the way to increase participation.

This type of nonrandom coverage bias resulting from low participation of women in mobile phone surveys can affect their representativeness, and such findings are important to document so that mobile phone surveys can be designed appropriately to weight survey samples by demographic factors that are reflective of the broader population. Previous studies have found that prenotification mechanisms can be promising [45], and examining different “primers,” such as SMS text messaging, a motivational call, or use of financial incentives to increase response rates among under-represented groups such as women, older participants, and rural participants needs further investigation. Our group previously found evidence to suggest that incentives may increase survey participation and reduce refusals [21]. Reasons for the observed differences and similarities between Bangladesh and Tanzania and implications for low and middle-income countries will warrant more examination in future studies. There may be minimal ethical concern as we asked very little sensitive information (ie, gender or age) and did not ask most identification questions (eg, name, date of birth, parental name, or address).

The surveys were conducted as long as the total number of required “complete interviews” (ie, desired sample size) was obtained; therefore, due to lower response rates, a higher number of phone calls was required for IVR. This is less likely to introduce any bias.

### Study Strengths and Limitations

The study reported in this paper was nested in another study whose primary purpose was to analyze consistency and reliability of survey responses using 2 different modes [26]. The original sample was powered for the parent study, not this study. However, collecting survey data contemporaneously using the same content but 2 different modes from the same target population has enabled this analysis of differential effects of mode on survey participation. Although the study leveraged an innovative random digit dialed sampling method to identify survey participants through their mobile phone numbers, there may have been delays in recruitment due to phones being turned off or without battery and issues with network coverages. Individual-level factors such as not being able to hear the phone ring or being busy, in addition to hanging up before consenting, may all have accounted for some of the nonresponse among those with existing mobile phone numbers whose status remained unknown. These factors may be amplified among rural residents, women, and other subgroups who may face multiple work and domestic responsibilities and the possible reluctance to take calls from unknown numbers. Second, an IVR-naïve population who may be unfamiliar with answering surveys may have contributed to lower response and completion rates. We mitigated some of these challenges by making 3 additional call-back attempts, randomly distributing the time of day for placing both the CATI and IVR surveys, and placing calls between 8 AM and 8 PM local time.

It is important to acknowledge the possibility of frame bias, that individuals reached through a mobile phone may not be representative of the broader population, and as such, our study findings may not be generalizable to subgroups of the population, particularly women, older adults, the poor, and respondents residing in rural locations who are generally considered under-represented in public health. We should note that it was not the objective of this study to produce nationally representative results and our findings should be interpreted with the appropriate caution as not necessarily being representative of these 2 countries. Multiple factors, including desired target population, may need to be considered in selecting which modality to use in a particular country. Furthermore, we did not check whether the voice of interviewer and survey mode had any interaction to obtain differences in participation. Finally, we recognize that participants may own multiple mobile phones, and thus, theoretically, some individuals could have answered the survey twice—although the likelihood of this is infinitesimally small [15] and unlikely to influence the results in a measurable way.

### Conclusions

After comparing 2 survey modes, we observed that using the CATI surveys generated higher survey response, cooperation, and completion rates than IVR surveys. However, for both survey modalities, notable gender, residence, education, and age differences exist among respondents of mobile phone surveys. Nevertheless, mobile phone surveys may complement existing survey methods and potentially be adapted to collect risk factor data on public health topics. We found that IVR surveys generally reached younger, more educated, and urban respondents. Although attractive due to their fully automated nature and relative ease of deployment, caution is needed in designing IVR surveys to ensure adequate representation of all segments of the population. Meanwhile, CATI surveys may offer a promising interim approach for surveying potentially under-represented groups like women, rural residents, and participants with lower levels of education in some countries.

## Appendix

Multimedia Appendix 1 Supplementary tables.

## Abbreviations

AOR: adjusted odds ratio
CATI: computer-assisted telephone interview
IRB: institutional review board
IVR: interactive voice response
UOR: unadjusted odds ratio

## Data Availability

All data files are available from the OpenICPSR repository database: accession number(s) ICPSR 107284.
