# Supplementary material for: Effect of the Data Collection Method on Mobile Phone Survey Participation in Bangladesh and Tanzania: Secondary Analyses of a Randomized Crossover Trial
Source: JMIR Form Res. 2023 Apr 20;7:e38774. doi: 10.2196/38774 (PMC10160933; doi:10.2196/38774)
Supplement: Multimedia Appendix 1 [file formative_v7i1e38774_app1.docx]

**Multimedia Appendix 1. Supplementary tables.**

**Table S1.** Equations used to calculate survey response rates.

| AAPOR^a^ category | | Equation ^b^ |
| --- | --- | --- |
| **Primary analysis (conservative scenario)** | | |
|  | Contact rate #1 | ($\left( I+P \right)+R+O)/$($\left( I+P \right)+R+O+NC+ (UH+UO))$ |
|  | Response rate #2 | ($I+P)/$($\left( I+P \right)+\left( R+O+ NC \right)+ (UH+UO))$ |
|  | Refusal rate #1 | *R*/($\left( I+P \right)+\left( R+O+NC \right)+ (UH+UO))$ |
|  | Cooperation rate #2 | I/($I+P+R+O)$ |
| **Secondary analysis (realistic scenario)** | | |
|  | Contact rate #2 | ($\left( I+P \right)+R+O)/$($\left( I+P \right)+R+O+NC+ e(UH+UO))$ |
|  | Response rate #4 | ($I+P)/$($\left( I+P \right)+\left( R+O+ NC \right)+ e(UH+UO))$ |
|  | Refusal rate #2 | *R*/($\left( I+P \right)+\left( R+O+NC \right)+ e(UH+UO))$ |
|  | Cooperation rate #1 | (I+P)/($I+P+R+O)$ |

^a^AAPOR: American Association for Public Opinion Research.

^b^I: interview; P: partial interview; R: refusal/call disconnected; O: other; NC: noncontact; UH: unknown household/phone number; UO: unknown other; *e*: estimated proportion of unknown cases expected to be age-eligible.

**Table S2.** Operational definitions of survey outcomes used in the study.

| AAPOR^a^ measure | Operational definition |
| --- | --- |
| Complete interview (I) | A respondent that answered all applicable questions |
| Partial interview (P) | A respondent who consented to the survey, answered at least 1 NCD^b^ question but did not answer all questions |
| Refusal (R) | A respondent who (1) did not consent to the survey or (2) formally withdrew during the survey |
| Disconnected call: eligible | Age-eligible and consented but the call was disconnected before any NCD module |
| Unknown eligibility (UO) | A respondent with a working phone number who answered the call but did not engage with the survey or answered the call, listened to the intro or provided other response but disconnected or hung-up before answering the age-eligibility questions |
| Unknown other (UO) | Phone exists but eligibility could not be determined because the respondent did not pick up the phone or the phone had busy tone, status unknown |
| Unknown household or phone number (UH) | A phone number for which the working status could not be determined (phone did not connect) |
| Ineligible | A respondent who answered the survey but was younger than 18 years old |
| *e* (estimated eligible) | Proportion of unknown cases that were estimated to be age-eligible |

^a^AAPOR: American Association for Public Opinion Research.

^b^NCD: noncommunicable disease.

**Table S3.** Secondary response rates by mobile phone survey delivery mode in Bangladesh and Tanzania.

| AAPOR^a,b^ category | Bangladesh | | Tanzania | |
| --- | --- | --- | --- | --- |
|  | CATI^c^ (n=1334), n (%) | IVR^d^ (n=1586), n (%) | CATI (n=787), n (%) | IVR (n=1402), n (%) |
|  |  |  |  |  |
| Contact rate #2 | 949 (71.2) | 783 (49.4) | 443 (56.3) | 700 (49.9) |
| Response rate #4 | 377 (28.3) | 498 (31.4) | 376 (47.8) | 586 (41.8) |
| Refusal rate #2 | 572 (42.9) | 285 (18.0) | 67 (8.5) | 114 (8.1) |
| Cooperation rate #1 | 359 (37.8)^e^ | 371 (47.4)^f^ | 375 (84.7)^g^ | 448 (64.0)^h^ |

^a^AAPOR: American Association for Public Opinion Research.

^b^Rates and numbers indicate key survey rates as defined by the AAPOR.

^c^CATI: computer-assisted telephone nterviews.

^d^IVR: interactive voice response.

^e^n=949.

^f^n=783.

^g^n=443.

^h^n=700.

**Table S4.** Odds ratios (ORs) from the multilevel logistic regression modeling (generalized linear latent and mixed models) for mode effects on secondary survey response rates for computer-assisted telephone interviews (CATIs) and interactive voice response (IVR) mobile phone surveys in Bangladesh and Tanzania.

| Dependent variable and covariates^a^ | | Bangladesh | | Tanzania | |
| --- | --- | --- | --- | --- | --- |
|  | | Adjusted OR (95% CI) | *P* value | Adjusted OR (95% CI) | P value |
|  | |  |  |  |  |
| **AAPOR**^b^ **response #4**^c,d,e^ | | | | | |
|  | IVR (reference: CATI)^f^ | 0.12 (0.07-0.20) | <.001 | 0.28 (0.14-0.56) | <.001 |
|  | Older age (50-69 and ≥70 years; reference:18-49 years) | 1.02 (0.48-2.13) | .97 | 0.85 (0.25-2.88) | .80 |
|  | Female (reference: male) | 0.52 (0.33-0.81) | .004 | 0.88 (0.44-1.77) | .72 |
|  | Rural (reference: urban) | 1.36 (0.94-1.98) | .10 | 1.49 (0.75-2.95) | .26 |
|  | Lower level of education (none or primary only; reference: at least secondary) | 9.37 (4.84-18.13) | <.001 | 0.85 (0.44-1.63) | .62 |
|  | _cons | 18.20 (10.36-31.97) | <.001 | 74.65 (33.17-167.98) | <.001 |
| **AAPOR cooperation #1**^d,g,h^ | | | | | |
|  | IVR (reference: CATI)^f^ | 0.09 (0.06-0.14) | <.001 | 0.09 (0.06-0.14) | <.001 |
|  | Older age (50-69 and ≥70 years; reference:18-49 years) | 0.71 (0.43-1.16) | .17 | 0.65 (0.35-1.20) | .17 |
|  | Female (reference: male) | 0.58 (0.41-0.84) | .003 | 0.74 (0.52-1.05) | .09 |
|  | Rural (reference: urban) | 1.45 (1.09-1.93) | .01 | 0.96 (0.69-1.35) | .83 |
|  | Lower level of education (none or primary only; reference: at least secondary) | 1.36 (1.01-1.83) | .04 | 0.75 (0.54-1.05) | .09 |
|  | _cons | 11.48 (7.35-17.93) | <.001 | 41.22 (24.82-68.45) | <.001 |

^a^All equations for fixed effects: IVR Older Female Rural LowerSchool _cons.

^b^AAPOR: American Association for Public Opinion Research.

^c^Bangladesh: level 1 n=1453 and level 2 n=5 mobile network providers; Tanzania: level 1 n=1493; level 2 n=4 mobile network providers with 8 plans used by respondents across the study rounds.

^d^Rates and numbers indicate key survey rates as defined by the AAPOR.

^e^Bangladesh: variances and covariances of random effects level 2 (network) var(1): 0.1084836 (0.10830973); Tanzania: variances and covariances of random effects level 2 (network) var(1): 0.17189969 (0.19861113).

^f^In this analysis, IVR was used in the first contact and is the primary survey mode; CATI used in the first contact is the counterfactual.

^g^Bangladesh: level 1 n=1453 and level 2 n=5 mobile network providers; Tanzania: level 1 n=752; level 2 n=4 mobile network providers with 8 plans used by respondents across the study rounds.

^h^Bangladesh: variances and covariances of random effects level 2 (network) var(1): 0.08334877 (0.08714233); Tanzania: variances and covariances of random effects level 2 (network) var(1): 0.0713261 (0.0681514).
